# Engineered Metal Oxide Nanoparticles as Fungicides for Plant Disease Control

**DOI:** 10.3390/plants12132461

**Published:** 2023-06-27

**Authors:** Aida R. Cruz-Luna, Alfonso Vásquez-López, Hugo Rojas-Chávez, Manuel A. Valdés-Madrigal, Heriberto Cruz-Martínez, Dora I. Medina

**Affiliations:** 1 Instituto Politécnico Nacional, CIIDIR-OAXACA, Hornos Núm 1003, Col. Noche Buena, Santa Cruz Xoxocotlán, Oaxaca 71230, Mexico; luna_060877@hotmail.com (A.R.C.-L.); avasquez@ipn.mx (A.V.-L.); 2 Tecnológico Nacional de México, Instituto Tecnológico de Tláhuac II, Camino Real 625, Alcaldía Tláhuac, Ciudad de México 13550, Mexico; rojas_hugo@ittlahuac2.edu.mx; 3 Tecnológico Nacional de México, Instituto Tecnológico Superior de Ciudad Hidalgo, Av. Ing. Carlos Rojas Gutiérrez 2120, Fracc. Valle de la Herradura, Ciudad Hidalgo 61100, Mexico; mavm8405@hotmail.com; 4 Tecnológico Nacional de México, Instituto Tecnológico del Valle de Etla, Abasolo S/N, Barrio del Agua Buena, Santiago Suchilquitongo, Oaxaca 68230, Mexico; 5 Tecnologico de Monterrey, Institute of Advanced Materials for Sustainable Manufacturing, Monterrey 64849, Mexico

**Keywords:** shape, size, sustainable agriculture, crop protection, antifungal activities

## Abstract

Metal oxide nanoparticles are considered to be good alternatives as fungicides for plant disease control. To date, numerous metal oxide nanoparticles have been produced and evaluated as promising antifungal agents. Consequently, a detailed and critical review on the use of mono-, bi-, and tri-metal oxide nanoparticles for controlling phytopathogenic fungi is presented. Among the studied metal oxide nanoparticles, mono-metal oxide nanoparticles—particularly ZnO nanoparticles, followed by CuO nanoparticles —are the most investigated for controlling phytopathogenic fungi. Limited studies have investigated the use of bi- and tri-metal oxide nanoparticles for controlling phytopathogenic fungi. Therefore, more studies on these nanoparticles are required. Most of the evaluations have been carried out under in vitro conditions. Thus, it is necessary to develop more detailed studies under in vivo conditions. Interestingly, biological synthesis of nanoparticles has been established as a good alternative to produce metal oxide nanoparticles for controlling phytopathogenic fungi. Although there have been great advances in the use of metal oxide nanoparticles as novel antifungal agents for sustainable agriculture, there are still areas that require further improvement.

## 1. Introduction

Agriculture is considered to be the backbone of countries around the world. However, it is plagued with numerous global challenges [1,2]. For instance, diseases caused by nematodes [3], bacteria [4], fungi [5,6], and other pathogens present in the environment cause large losses of crops. In particular, phytopathogenic fungi produce various types of diseases in economically important crops, directly impacting the world economy [7,8]. They can also affect the different stages of a crop (e.g., sowing, growth, production, and postharvest) [9,10]. Therefore, it is essential to control these microorganisms.

Currently, phytopathogenic fungi can be controlled using agrochemical products, which are cheap and easily available on the market. However, these chemicals have not only negatively affected both soil and air, but have also led to the eutrophication of water bodies worldwide [11,12]. Thus, many researchers have proposed novel, ingenious, and ecofriendly alternatives for controlling phytopathogenic fungi in agriculture.

Recently, different environmentally friendly and efficient alternatives have been proposed to control phytopathogenic fungi, such as plant extracts [13], biological control [14], essential oils [15], and engineered nanomaterials [16]. Among these alternatives, the use of nanomaterials has been the most explored. Engineered nanomaterials have gained great importance for controlling phytopathogenic fungi, owing to their different physicochemical properties compared to their bulk counterparts [16]. Consequently, various nanomaterials have shown better results than conventional agrochemicals for plant disease control [17].

To date, different types of nanomaterials have been explored as alternatives for controlling phytopathogenic fungi, such as nanopolymers [18], carbon nanomaterials [19], and metal nanoparticles [20,21,22,23]. In particular, metal oxide nanoparticles are considered to be an efficient and ecofriendly alternative for controlling phytopathogenic fungi in agriculture [24,25,26,27,28,29,30,31,32,33].

Today, there are several review articles on the use of nanomaterials in sustainable agriculture. However, these reviews generally explore various types of nanomaterials (e.g., nanopolymers, nanocarbons, metal nanoparticles) and applications (e.g., fertilizers, nematicides, bactericidal, fungicides) in agriculture, where the use of metal oxide nanoparticles for controlling phytopathogenic fungi is not analyzed in detail [24,25,26,27,28,29,30,31]. In other cases, the reviews are focused on the use of metal oxide nanoparticles forming composite materials for the control of phytopathogenic fungi [32]. Therefore, to date, there is a lack of review articles—critical and detailed—on the current progress of metal oxide nanoparticles for controlling phytopathogenic fungi. Therefore, this review discusses and analyzes the role of mono-, bi-, and tri-metal oxide nanoparticles for controlling phytopathogenic fungi in sustainable agriculture. Furthermore, this review article provides the challenges and future directions regarding the application of metal oxide nanoparticles as potential antifungal agents in sustainable agriculture.

## 2. Antifungal Properties of Mono-Metal Oxide Nanoparticles

### 2.1. Zinc Oxide Nanoparticles

**Figure 1:**
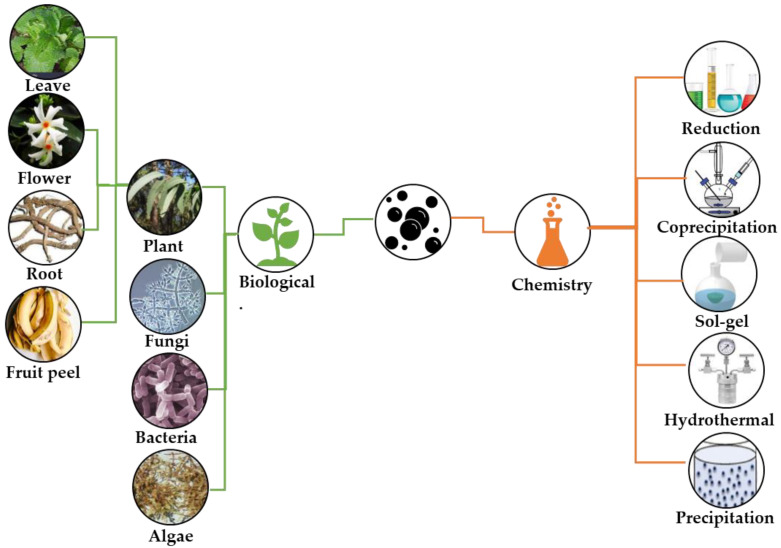
Synthesis methods used to produce ZnO-NPs for the control of phytopathogenic fungi in agriculture.

Zinc oxide nanoparticles (ZnO-NPs) have wide applications in different fields owing to their excellent characteristics, including cost-effectiveness, ease of manufacture, chemical stability, and non-toxicity [28,29,30,34,35,36,37]. In agriculture, there have been many studies on the use of ZnO-NPs as novel antifungal agents, with promising results [38,39,40,41,42,43,44,45,46,47,48,49,50,51,52,53,54,55,56,57,58,59,60,61,62,63,64,65,66,67,68,69,70,71,72,73,74,75,76,77,78,79,80,81,82,83,84,85,86]. These nanoparticles were prepared using biological [38,39,43,44,45,46,47,48,49,50,51,52,53,54,55,56,57,58,59,60,61,64] and chemical syntheses [62,63,65,66,67,68,69,70,71,72,73,74,75]. Figure 1 illustrates the different synthesis methods used to produce ZnO-NPs. For biological synthesis, different extracts from plants and microorganisms have been used (Figure 1), while for chemical synthesis, easy and inexpensive synthesis routes have been employed (Figure 1). Biological routes are more used than chemical routes for the synthesis of ZnO-NPs to control phytopathogenic fungi, because this is an environmentally friendly approach [30].

It has been reported that the characteristics of nanoparticles (e.g., shape, size distribution, crystallinity, composition, crystalline phase, surface chemistry, and agglomeration) substantially determine their antifungal activities [87,88,89], which can be controlled based on the methods and conditions of synthesis. Therefore, several studies have analyzed the effects of the methods and conditions of synthesis on the characteristics of ZnO-NPs for their application as antifungal agents in agriculture [90,91,92,93]. For instance, a previous study investigated the effects of green (using *Aloe vera* plant extract) and chemical synthesis on the size and shape of ZnO-NPs [92]. The authors reported that the average size of ZnO-NPs synthesized by chemical and biological routes was 75 nm and 95 nm, respectively. In addition, ZnO-NPs obtained from chemical synthesis were spherical, while those obtained by biological routes were hexagonal. They also showed that ZnO-NPs obtained by chemical routes were more effective against *Alternaria solani* than the ZnO-NPs obtained using the biological route [92]. In another study, ZnO-NPs synthesized by chemical routes showed good crystallinity and a spheroidal shape, while ZnO nanobiohybrids obtained by a green route presented low crystallinity and a laminar morphology [93]. The ZnO-NPs obtained by chemical routes caused the highest percentage inhibition against *Cercospora* sp.

**Figure 2:**
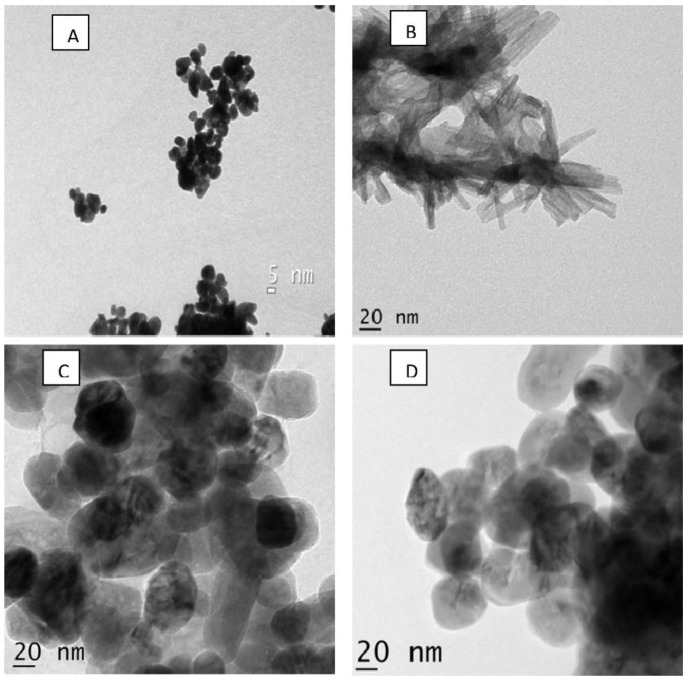
Transmission electron micrographs of four different ZnO-NPs: (**A**) *Beta vulgaris*, (**B**) *Cinnamomum Tamala*, (**C**) *Cinnamomum verum*, and (**D**) *Brassica oleracea*. Reproduced from reference [47] with permission from Elsevier.

Different biological synthesis conditions and extracts (from plants and organisms) have also been evaluated to produce ZnO-NPs for controlling phytopathogenic fungi [43,47,53,55,58]. In recent years, different extracts (e.g., *Beta vulgaris*, *Cinnamomum tamala*, *Cinnamomum verum*, and *Brassica oleracea*) have been used to produce ZnO-NPs [47]. As shown in Figure 2, the type of extract also affected the size and shape of the ZnO-NPs. ZnO-NPs prepared using *Beta vulgaris* and *Brassica oleracea* were found to be active against *Aspergillus niger*, showing that the characteristics of the ZnO-NPs substantially influence their antifungal activity. More recently, ZnO-NPs were obtained using aqueous extracts of different seaweeds (e.g., *Ulva lactuca* and *Solanum marginatum*). The sizes of the nanoparticles synthesized using *Ulva lactuca* and *Solanum marginatum* were in the range of 12–17 nm and 6–11 nm, respectively [58]. ZnO-NPs synthesized using *Solanum marginatum* showed the best results against various species of phytopathogenic fungi. In another study, ZnO-NPs were synthesized using *Cinnamomum camphora* leaf extracts with different pH values (i.e., 7, 8, and 9), and their effects against *Alternaria alternata* were evaluated [53]. The average sizes of the ZnO-NPs synthesized at pH 7, pH 8, and pH 9 were about 13.92 nm, 15.19 nm, and 21.13 nm, respectively. ZnO-NPs at pH 7 were found to be spherical, but they showed irregular spherical shapes when the pH value increased. The nanoparticles of 13.92 nm and spherical shape (synthesized at pH 7) showed the best antifungal activity compared to the other nanoparticles synthesized at other pH values. Recently, ZnO-NPs were obtained through a biological approach using *Justicia adhatoda* leaf extracts with different precursors (e.g., zinc sulfate, zinc nitrate, and zinc acetate dihydrate) [55]. By varying the type of metal precursor, different sizes and shapes of ZnO-NPs were obtained. For example, ZnO-NPs synthesized from a zinc sulfate precursor were orthogonal/nanorod, with an average diameter of ~30 nm. The ZnO-NPs synthesized from zinc sulfate showed the best antifungal activity against *Aspergillus niger* and *Aspergillus fumigatus*.

Different chemical synthesis methods and conditions have been evaluated to produce ZnO-NPs for the control of phytopathogenic fungi [62,65,66,67,68,71]. For example, ZnO-NPs were synthesized with and without surfactants in [62]. The nanoparticles obtained without a surfactant were larger than those synthesized with surfactants. Moreover, ZnO-NPs obtained without a surfactant presented better antifungal activities than those synthesized with surfactants [62]. In another study, ZnO-NPs of different sizes, shapes, and states of agglomeration were produced with different concentrations of the precursor and different volumes of the solvent [65]. ZnO-NPs synthesized with 13.17 g of zinc acetate dihydrate in 400 mL of ethanol presented two types of morphology (i.e., spherical and acicular) and sizes between 20 and 35 nm, while nanoparticles produced from the same amount of metal precursor but dissolved in 600 mL of ethanol were spherical nanoparticles with sizes between 30 and 45 nm. ZnO-NPs synthesized in 400 mL of ethanol presented better antifungal activities against *Erythricium salmonicolor* than those synthesized in 600 mL of ethanol. Recently, ZnO-NPs were synthesized using the coprecipitation and hydrothermal methods of chemical synthesis [66]. The average size of the ZnO-NPs obtained by the coprecipitation method was smaller than that of those formed by the hydrothermal procedure [66]. ZnO-NPs synthesized exhibited good antifungal activity results against *Colletotrichum gloeosporioides*. In another study, the precipitation method with different synthesis conditions was used to produce ZnO-NPs that were either rod or spheroidal structures [67]. ZnO-NPs with a rod shape had a higher antifungal efficiency than those with a spheroidal shape. More recently, colloidal and hydrothermal routes were used to produce spheroidal, platelet, and rod morphologies of ZnO-NPs [71]. The diameters of the spheroidal, platelet, and rod structures were 18 ± 4, 246 ± 40, and 786 ± 142 nm, respectively. Moreover, ZnO structures with a platelet shape presented better antifungal activities than the other two structures against three species of fungi (*Fusarium oxysporum*, *Fusarium solani*, and *Colletotrichum gloeosporioides*). For both synthesis routes (e.g., chemical and biological), the different characteristics of the nanoparticles, obtained by modifying the synthesis conditions and methods, directly influenced their antifungal activities.

**Figure 3:**
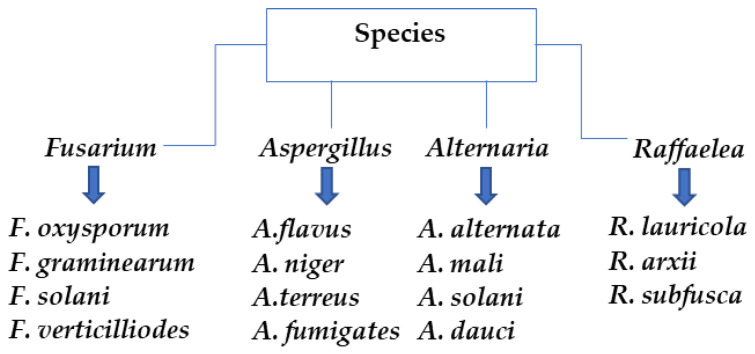
The most common fungal species evaluated using ZnO-NPs.

Research works have also showed that other factors, such as the concentration of nanoparticles used to inhibit the growth of phytopathogenic fungi and characteristics of fungal species, influence the antifungal activity [87]. In general, the inhibition of phytopathogenic fungi tends to increase under in vitro evaluations when the concentration of the ZnO-NPs increases [46,48,49,50,51,53,54,60,63,64,65,66,67,69,70,71,72,73,74,75,76,77,78,79,80,81,82,83,84,85]. Interestingly, low concentrations (100–1000 ppm) of ZnO-NPs have shown excellent results for controlling phytopathogenic fungi. Moreover, the antifungal activity of ZnO-NPs has been studied against different species of phytopathogenic fungi [44,48,49,50,52,54,55,58,70,71,72,73,75,77,78,81,84,85]. It was observed that the morphological and physiological characteristics of fungal species affect the inhibition properties of the ZnO-NPs. Figure 3 illustrates the most common fungal species evaluated. Most of these evaluations have been carried out under in vitro conditions. Fortunately, there have been some studies carried out under in vivo conditions [42,51,54,74,83,84,85,86]. For instance, ZnO-NPs were tested against *A. alternata* in tomato fruit (*Lycopersicon esculentum* cv mojito) with uniform maturity, shape, and size [42]. In another study, ZnO-NPs obtained from olive leaf extracts were evaluated against *B. cinerea* in faba bean plants (*Vicia faba* major L.) [51]. Moreover, synthesized ZnO-NPs were evaluated against *R. solani*, *Fusarium* sp., and *M. phaseolina* on cotton cultivars [54]. In another study, ZnO-NPs were evaluated against *F. oxysporum* in tomato plants (*S. lycopersicum*) [74]. These studies demonstrated the favorable role of ZnO-NPs for plant disease control [42,51,54,74,83,84,85,86].

**Figure 4:**
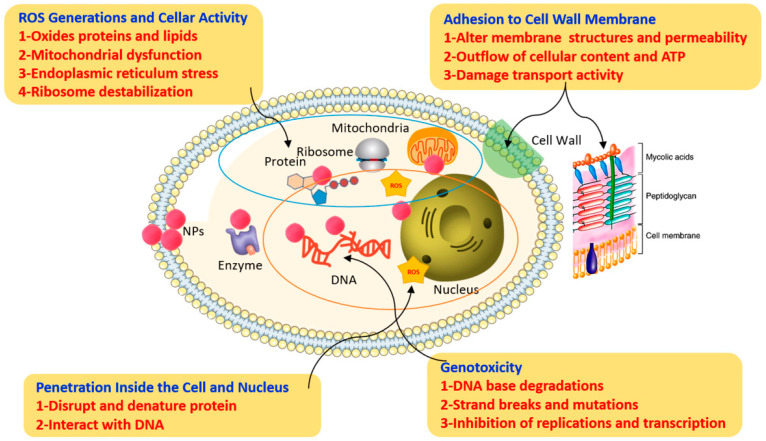
Possible antifungal action mechanisms of metal oxide nanoparticles. Reproduced from reference [32].

As previously mentioned, there are several factors (e.g., characteristics of nanoparticles, the concentration of nanoparticles used to control phytopathogenic fungi, morphological and physiological characteristics of fungal species) that influence the antifungal activity of metal oxide nanoparticles. Therefore, various action mechanisms of the metal oxide nanoparticles on the phytopathogenic fungi have been proposed [32,87,88]. Figure 4 illustrates the different possible antifungal action mechanisms of these nanoparticles.

### 2.2. Copper Oxide Nanoparticles

Copper oxide nanoparticles (CuO-NPs) have numerous applications in medicine, agriculture, catalysis, cosmetics, and electronics, among others [29,94,95]. In agriculture, CuO-NPs have been widely used to inhibit the growth of phytopathogenic fungi [38,52,73,80,81,86,95,96,97,98,99,100,101,102,103,104,105]. These nanoparticles are produced mainly by biological methods [38,52,73,96,97,98,99,100]. In addition, the antifungal properties of commercial CuO-NPs have also been evaluated [80,81,86,101,102,103,104]. Various biological synthesis conditions and extracts (from plants and organisms) have also been employed to produce CuO-NPs for the control of phytopathogenic fungi [52,73,96,97,98,99,100]. The type of extracts used in the biosynthesis affects the size of the CuO-NPs, and most of the different types of extracts produce spherical nanoparticles [52,73,96,97,98,99,100]. As previously mentioned, the concentration of nanoparticles and the species of the fungi are important factors that influence antifungal activity [87]. As in the case of ZnO-NPs, the antifungal activity of CuO-NPs tends to increase with the increase in the nanoparticles’ concentration [73,80,81,96,97,100]. Interestingly, low concentrations (100–1000 ppm) of CuO-NPs have shown good results in the control of phytopathogenic fungi. Moreover, the effects of CuO-NPs on different species of phytopathogenic fungi have been evaluated [52,73,81,96,98,100]. The morphological and physiological characteristics of fungal species have also been found to have an important effect on the inhibition properties of CuO-NPs [52,73,81,96,98,100].

### 2.3. Iron oxide Nanoparticles

Iron oxide nanoparticles are also widely used in different fields [106,107,108]. Some studies have evaluated the effects of these metal oxides on phytopathogenic fungi [70,81,97,101,109,110,111,112]. Interestingly, biological synthesis is widely used to produce these nanoparticles [97,109,111,112]. For instance, iron oxide (Fe_2_O_3_) nanoparticles obtained using leaf extracts of *Euphorbia helioscopia* had a spherical shape and were in the range of 7–10 nm in size. These oxides showed promising and better results than CuO against *Cladosporium herbarum* [97]. In another study, iron oxide (Fe_2_O_3_ and Fe_3_O_4_ mixed phase) nanoparticles with size of 10–30 nm were synthesized using tannic acid, and their effects in inhibiting the growth of *Trichothecium roseum, Cladosporium herbarum, Penicillium chrysogenum, Alternaria alternata,* and *Aspergillus niger* were evaluated [109]. These nanoparticles exhibited significant activities against all of the tested fungal agents. Moreover, the inhibition activity of the fungal agents increased with the increase in the concentration of these nanoparticles [109]. Recently, iron oxide (Fe_2_O_3_) nanoparticles with a size of 207 ± 2 nm were synthesized using *Trichoderma harzianum* and evaluated against *Sclerotinia sclerotiorum.* These nanoparticles showed their potential for controlling *Sclerotinia sclerotiorum* [111]. Finally, iron oxide (Fe_2_O_3_) nanoparticles synthesized using *Aegle marmelos* extract showed promising results when evaluated against *Fusarium solani* [112].

### 2.4. Magnesium Oxide Nanoparticles

Magnesium oxide nanoparticles (MgO-NPs) are another type of metal oxide investigated in the control of phytopathogenic fungi [63,70,110,113,114,115]. Commercial MgO-NPs and those synthesized by chemical and biological routes are widely used. MgO-NPs have been prepared using *Carica papaya* leaf extract and evaluated against *Phytophthora nicotianae* and *Thielaviopsis basicola* under laboratory and greenhouse conditions [113]. These nanoparticles showed promising results for controlling phytopathogenic fungi. Recently, MgO-NPs with a size of 15 ± 4 nm showed promising results when they were evaluated against various phytopathogenic fungi [115].

### 2.5. Titanium Oxide Nanoparticles

**Figure 5:**
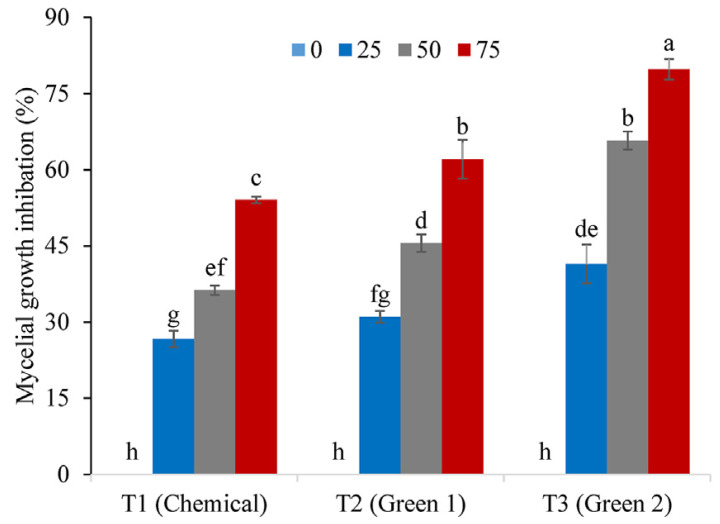
Antifungal activities of different concentrations of TiO_2_-NPs prepared by different methods (T1: synthesized by the sol–gel method, T2: synthesized using *Trianthema portulacastrum*, and T3; synthesized using *Chenopodium quinoa*). Reproduced from reference [116] with permission from Elsevier.

The role of titanium oxide nanoparticles for controlling different species of phytopathogenic fungi has also been evaluated [80,84,101,102,116,117,118]. As in the previous cases, commercial nanoparticles [80,84,101,102] and those obtained by chemical [116,117] and biological [116,118] routes have been evaluated to inhibit the growth of different pathogens. In one study, titanium oxide (TiO_2_-NPs) nanoparticles obtained by biological and chemical routes were evaluated against *Ustilago tritici* [116]. With respect to chemical synthesis, TiO_2_-NPs were synthesized by the sol–gel method (T1), while for the biological route, TiO_2_-NPs were synthesized using plant extracts of *Trianthema portulacastrum* (T2) and *Chenopodium quinoa* (T3). The type of synthesis method and the type of extract used determined the size of the nanoparticles. Three concentrations (25 μL, 50 μL, and 75 μL of 0.10 mg mL^−1^) of all synthesized TiO_2_-NPs were evaluated against *Ustilago tritici*, as shown in Figure 5. TiO_2_-NPs T2 and T3 presented better results than those synthesized by the chemical route, and T3 (*Chenopodium quinoa*) exhibited the best results of the synthesized nanoparticles, as shown in Figure 5.

### 2.6. Other Types of Mono-Metal Oxide Nanoparticles

**Figure 6:**
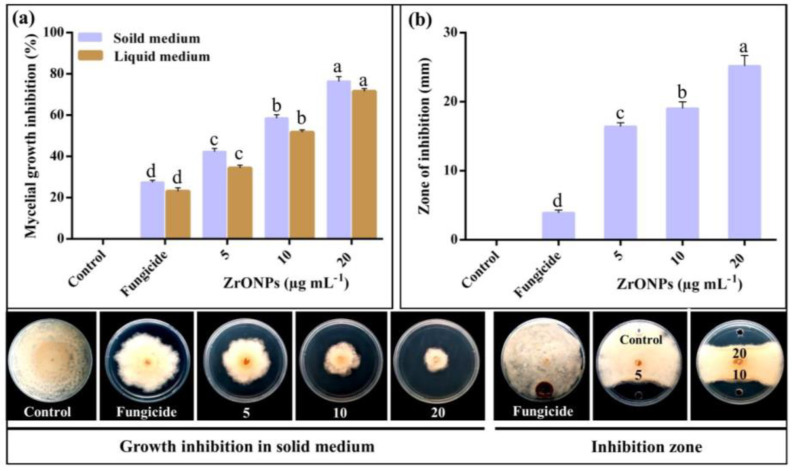
In vitro antifungal activity of ZrO-NPs at various concentrations against *Pitiriasis versicolor*: (**a**) Mycelial growth inhibition on solid and liquid media. (**b**) Inhibition zone was determined using well diffusion assay. Reproduced from reference [120] with permission from Elsevier.

**Figure 7:**
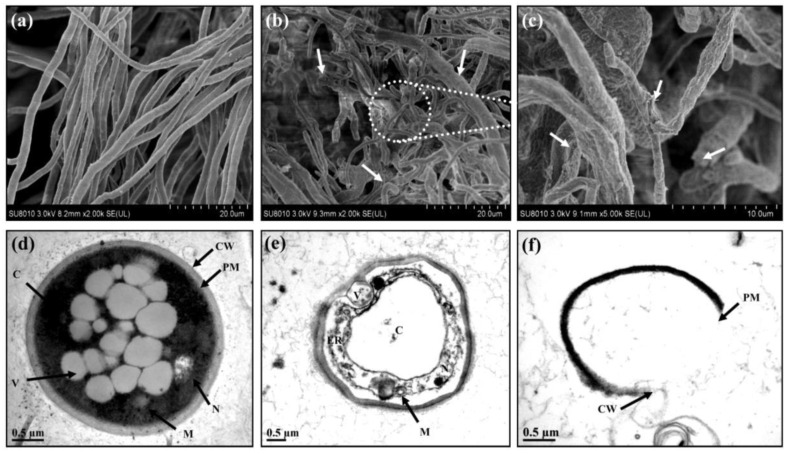
Scanning electron microscope (SEM) and transmission electron microscope (TEM) images of *Pitiriasis versicolor*: (**a**,**d**) *Pitiriasis versicolor* cells treated with sterile water; (**b**,**c**) *Pitiriasis versicolor* cells treated with 20 μg mL^−1^ ZrO-NPs demonstrated a highly damaged hyphal structure; (**e**,**f**) *Pitiriasis versicolor* cells treated with 20 μg mL^−1^ ZrO-NPs showed integrated cell wall and plasma membrane, disorganized cytoplasm, and damaged cell organelles. Note: CW = cell wall; PM = plasma membrane; N = nucleus; V = vacuoles; M = mitochondrion; C = cytoplasm; ER = endoplasmic reticulum. Reproduced from reference [120] with permission from Elsevier.

There are other types of oxide nanoparticles whose roles in inhibiting the growth of different species of phytopathogenic fungi have been studied; these include zirconium, [119,120,121], silicon [80,84], and manganese [80,86] oxide nanoparticles. Interestingly, zirconium nanoparticles (ZrO-NPs) have been produced using biological methods, and their effects against various phytopathogenic fungi were subsequently evaluated [120,121]. ZrO-NPs were produced using biological synthesis and evaluated against *Pestalotiopsis versicolor* [120]. The obtained ZrO-NPs had spherical shapes, in the range of 33–75 nm in size, and revealed a higher inhibition of the mycelium growth of *Pitiriasis versicolor* compared with the controls, as shown in Figure 6. As the concentration of ZrO-NPs increased, the inhibition of *Pitiriasis versicolor* also tended to increase. Moreover, the effect of ZrO-NPs on the fungal morphology was also analyzed. When *Pitiriasis versicolor* was exposed to ZrO-NPs at 20 μg mL^−1^ concentration, its hyphal structure exhibited substantial changes, as shown in Figure 7.

## 3. Antifungal Properties of Bi-Metal and Tri-Metal Oxide Nanoparticles

### 3.1. Bi-Metal Oxide Nanoparticles

**Figure 8:**
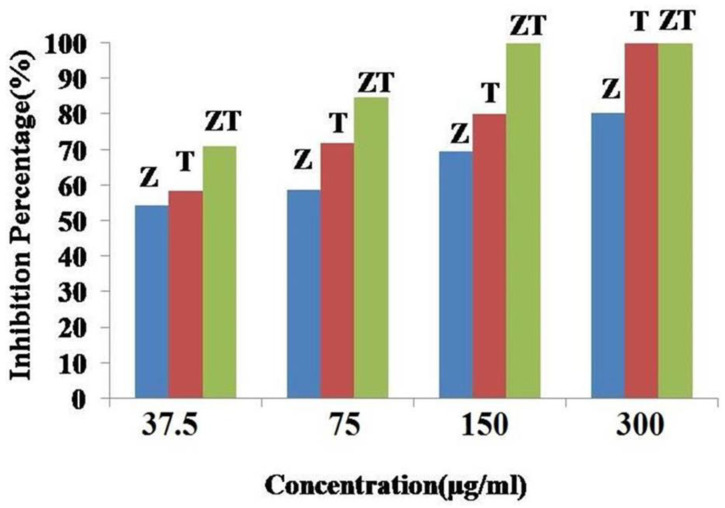
The fungicidal inhibition zones for ZnO (Z), TiO_2_ (T), and ZnO-TiO_2_ (ZT) nanoparticles against *Aspergillus flavus*. Reproduced from reference [117] with permission from Elsevier.

Bi-Metal oxide nanoparticles have different properties compared to mono-metal oxide nanoparticles. Therefore, they have gained great importance in different fields [122,123,124,125]. Several studies have examined the effects of bimetal oxide nanoparticles (e.g., ZnO-CuO, ZnO-MgO, ZnO-TiO_2_, ZnO-Mn_2_O_3_, ZnO-Mg(OH)_2_, CuO-Mn_2_O_3_) on the growth of phytopathogenic fungi and have reported their outstanding antifungal properties [66,86,117,126,127]. Some studies compared the antifungal activities of ZnO-MgO and ZnO-Mg(OH)_2_ nanoparticles synthesized by coprecipitation and hydrothermal methods with those of ZnO and MgO nanoparticles [66]. ZnO nanoparticles showed higher inhibition than MgO, ZnO-MgO, and ZnO-Mg(OH)_2_ nanoparticles. Therefore, the presence of MgO in bimetal oxide nanoparticles had a negative effect on antifungal activity against *Colletotrichum gloeosporioides* [66]. In another study, the antifungal activities of ZnO, TiO_2_, and ZnO-TiO_2_ nanoparticles were evaluated against *Aspergillus flavus* under in vitro conditions. ZnO-TiO_2_ nanoparticles exhibited higher antifungal activity against *Aspergillus flavus* than pure TiO_2_ and ZnO nanoparticles, as shown in Figure 8 [117]. These findings indicate that the formation of bi-metal oxide nanoparticles improved their antifungal activity against *Aspergillus flavus.*

### 3.2. Tri-Metal Oxide Nanoparticles

Recently, tri-metal systems were investigated for different applications because of their different properties compared to mono-metal and bi-metal systems [128,129,130]. This opened a great area of opportunity for the application of these ternary systems. Studies reported in the literature have examined the effects of tri-metal oxide nanoparticles (e.g., CuO-Mn_2_O_3_-ZnO) for controlling phytopathogenic fungi [86]. However, it is necessary to conduct more research on the use of these nanoparticles for the control of phytopathogenic fungi.

## 4. Challenges

Over the last few decades, engineered metal oxide nanoparticles have been studied and used for plant disease control. Based on this review, the following challenges are proposed:

1. Potential ecological effects: Engineered metal oxide nanoparticles, like any other chemical product, may pose environmental dangers through the leakage of nanoparticles into soil or water, impacting non-target organisms. Before these particles are widely used in agriculture or other industries, their possible environmental implications must be studied.
2. Inadequate efficacy: While designed metal oxide nanoparticles may have powerful antifungal characteristics, their effectiveness may vary depending on the type of fungus and environmental factors such as humidity, temperature, and pH. More research is needed to enhance their effectiveness against a variety of fungal infections.
3. Inadequate standardization: There are no defined techniques for the synthesis, characterization, and testing of tailored metal oxide nanoparticles as fungicides. The absence of uniformity makes comparing the results of different studies and drawing conclusions about their efficacy and safety difficult.
4. Resistance risk: As with most antifungal drugs, repeated use of tailored metal oxide nanoparticles as fungicides may result in the formation of resistant fungal strains. Strategies must be devised to reduce the possibility of resistance development while also extending the usefulness of these nanoparticles.
5. Concerns about toxicity: If engineered metal oxide nanoparticles penetrate the food chain or are swallowed directly, they may be harmful to humans and animals. Before these particles are widely used, their toxicity must be thoroughly investigated.

Finally, the use of metal oxide nanoparticles as fungicides has tremendous potential for reducing fungal diseases in crops and other environments. However, before widespread implementation, the potential problems and consequences must be carefully considered. To optimize their efficacy and safety, extensive research and standardization of techniques for their synthesis, characterization, and testing, as well as risk assessment, are required.

## 5. Future Directions

Metal oxide nanoparticles have various advantages as fungicides, including greater efficacy, less environmental impact, and lower application frequencies. Furthermore, the possible development of hybrid nanoparticles that mix two or more distinct metal oxides, such as copper oxide and zinc oxide, has the potential to provide synergistic benefits for increased antifungal activity.

The creation of innovative nanoparticles with increased stability, biocompatibility, and targeted distribution is among the future directions in the use of synthetic metal oxide nanoparticles as fungicides. Efforts are also being made to produce nanoparticles capable of activating plant defense mechanisms and promoting disease resistance. Combining metal oxide nanoparticles with biological control agents may also contribute to the creation of more effective and long-lasting control techniques for plant fungal infections.

## 6. Conclusions

To date, there have been notable advances in the use of metal oxide nanoparticles for controlling phytopathogenic fungi. These nanoparticles have shown promising results for the control of phytopathogenic fungi. However, most of these evaluations have been carried out under in vitro conditions. Among the studied metal oxide nanoparticles, mono-metal oxide nanoparticles are the most investigated nanoparticles for controlling phytopathogenic fungi, with promising results; in particular, ZnO-NPs are the most investigated for controlling phytopathogenic fungi, followed by CuO-NPs. There have been limited studies on the use of and tri-metal and bi-metal oxide nanoparticles for the control of phytopathogenic fungi. The results obtained in these studies are contradictory, because some studies suggest that these nanoparticles improve antifungal activity, while other studies conclude the opposite. Many biological and chemical synthesis methods have been used to produce metal oxide nanoparticles for controlling phytopathogenic fungi. However, these are mainly polydisperse in size and spherical in shape. Therefore, several challenges need to be addressed to obtain high-quality and efficient commercial products.

## Data Availability

Not applicable.
